# Genes expression profiles in vascular cambium of *Eucalyptus urophylla* × *Eucalyptus grandis* at different ages

**DOI:** 10.1186/s12870-023-04500-8

**Published:** 2023-10-18

**Authors:** Guo Liu, Zhihua Wu, Jianzhong Luo, Chubiao Wang, Xiuhua Shang, Guowu Zhang

**Affiliations:** 1 State Key Laboratory of Tree Genetics and Breeding, Chinese Academy of Forestry, Beijing, China; 2 Research Institute of Fast-Growing Trees, Chinese Academy of Forestry, Zhanjiang, China

**Keywords:** Transcriptome, Vascular cambium, *Eucalyptus urophylla* × *Eucalyptus grandis*, Different ages, Transcription factor, Phytohormone

## Abstract

**Background:**

Wood is a secondary xylem generated by vascular cambium. Vascular cambium activities mainly include cambium proliferation and vascular tissue formation through secondary growth, thereby producing new secondary phloem inward and secondary xylem outward and leading to continuous tree thickening and wood formation. Wood formation is a complex biological process, which is strictly regulated by multiple genes. Therefore, molecular level research on the vascular cambium of different tree ages can lead to the identification of both key and related genes involved in wood formation and further explain the molecular regulation mechanism of wood formation.

**Results:**

In the present study, RNA-Seq and Pac-Bio Iso-Seq were used for profiling gene expression changes in *Eucalyptus urophylla* × *Eucalyptus grandis* (*E. urograndis*) vascular cambium at four different ages. A total of 59,770 non-redundant transcripts and 1892 differentially expressed genes (DEGs) were identified. The expression trends of the DEGs related to cell division and differentiation, cell wall biosynthesis, phytohormone, and transcription factors were analyzed. The DEGs encoding expansin, kinesin, cycline, PAL, GRP9, KNOX, C2C2-dof, REV, etc., were highly expressed in *E. urograndis* at three years old, leading to positive effects on growth and development. Moreover, some gene family members, such as *NAC*, *MYB*, *HD-ZIP III*, *RPK*, and *RAP*, play different regulatory roles in wood formation because of their sophisticated transcriptional network and function redundantly.

**Conclusions:**

These candidate genes are a potential resource to further study wood formation, especially in fast-growing and adaptable eucalyptus. The results may also serve as a basis for further research to unravel the molecular mechanism underlying wood formation.

**Supplementary Information:**

The online version contains supplementary material available at 10.1186/s12870-023-04500-8.

## Background

Wood (secondary xylem) is a crucial energy resource on earth and can be used to replace fossil fuel resources in the future. Moreover, wood plays a vital role in sequestering atmospheric carbon [1]. All woody plants and some non-woody trees undergo primary and secondary growth [2]. The primary growth of trees consists of germination and flowering in the spring and the growth of branches, leaves, and roots during the growing season [3]. Secondary growth includes cambium differentiation and xylem formation. These are developmental processes driving the radial expansion of woody stems and are supported by the vascular cambium, which is a common lateral meristem in plants that produces secondary xylem and phloem [4, 5]. Tree stem thickening is accomplished by the meristematic activity of the vascular cambium [6]; therefore, the vascular cambium plays a critical role in wood formation and determines the yield, structure, and properties of wood [7]. Vascular cambium activities mainly include cambium proliferation and vascular tissue formation through secondary growth, thereby producing new secondary phloem inward and secondary xylem outward and leading to continuous tree thickening and wood formation. In particular, xylem cells, with thick secondary cell walls rich in lignin, cellulose and hemicellulose, are mainly responsible for transporting water, nutrients and minerals from the root to the rest of the plant [8]. Unlike xylem cells, phloem tissue is the main conduit for sugars in plants [9]. Therefore, molecular level research on the vascular cambium of different tree ages can lead to the identification of both key and related genes involved in wood formation and further explain the molecular regulation mechanism of wood formation. The findings can not only be applied to the improvement of wood properties, but also improve the purpose and operability of forest tree breeding.

Vascular cambium is an important tissue exists in forest trees, its activities are regulated by endogenous developmental programs and environmental cues and are highly complex and dynamic [10, 11]. Because of the rapid advancements in molecular biology, the molecular mechanisms that regulate cambium activity and related genes have been understood in the model plants of *Arabidopsis* and *Populus* [7]. Studies have reported that secondary wall biosynthesis genes [1, 12], cell division and differentiation genes [13], plant hormones [14–17], and transcription factors [5, 18–20] are also involved in the regulation of vascular cambium activities. The regulatory factors act both independently and by interacting with each other [21]. *Arabidopsis* serves as a model organism for studying cambium development and wood formation, with recent studies identifying some core genes and their regulatory mechanisms involved in cambium activity regulation. Rahimi et al. [22] discovered that *Arabidopsis* AHL15 transcriptional regulator plays a vital role in controlling vascular cambium activity. Sugimoto et al. [23] reported that *NTL9* negatively regulates *Arabidopsis* vascular cambium development during the secondary growth of the stem. Ben-Targem et al. [24] shed light on a pivotal hormone cross-talk between GA and auxin in plant secondary growth. Zhang et al. [5] highlighted the central roles of WUSCHEL-related homeobox 4 (WOX4) and PETAL LOSS in *Arabidopsis* cambium development. Several candidate genes involved in the initiation of vascular cambium formation have been identified in poplar. Zhu et al. [10] elucidated that the PtCLE20 peptide plays a crucial role in lateral growth regulation by repressing cambium activity in trees. Zheng et al. [25] revealed the role of vascular cambium-related two MADS-box genes (*VCM1* and *VCM2*) in the regulation of proliferation activity of the vascular cambium and secondary growth by modulating the subcellular auxin homeostasis in *Populus*. Kim et al. [8] and Zhu et al. [26] verified that *PtrHAM4–1* and *PtrHB4* overexpression significantly increased vascular cambium development in transgenic poplars.

*Eucalyptus* spp. is an important plantation species, which has been planted in 95 countries worldwide in an area exceeding 22.57 million ha [27]. Moreover, it is the most important and widely distributed forest tree species for industrial raw materials in southern China [28, 29]. According to the latest statistics, the total area of *Eucalyptus* plantations has reached 5.46 million ha in China [30]. Because of their wood quality and adaptation to diverse environmental conditions, *Eucalyptus* spp. and their interspecific hybrids have been increasingly adopted in forestry programs [31]. Especially, the hybridization of *E. urophylla* × *E. grandis* (*E. urograndis*) has provided excellent results in terms of wood quality and growth [32]. *E. urograndis* is a fast-growing tree with well-developed secondary vascular tissues, leading to its use in the evaluation of wood formation. Therefore, this study selected *E. urograndis* as the research material to reveal the transcriptional regulation and molecular mechanism of vascular cambium at different tree ages, thereby assisting in the identification of key genes responsible for the fast growth of *Eucalyptus* and providing new approaches to improve the biological and directional properties of wood in the artificial forest.

## Results

### Chemical composition analysis of vasular cambium

**Fig. 1:**
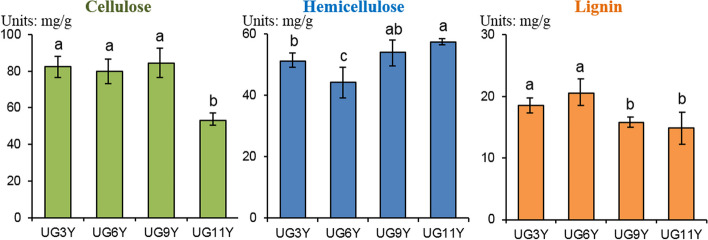
The results of three major main wood chemical compositions components of in the vascular cambium *E. urograndis* at different ages. The same letters in the figure indicate no significant difference, and the different letters indicate significant differences. The date was subjected to the method of multiple comparison of LSD. Error bars indicate standard error derived from three replicates

Wood is mainly composed of cellulose, hemicellulose, and lignin, which varies in proportion among different species. To analyze the chemical composition of *E. urograndis* at different ages, the cellulose, hemicellulose, and lignin contents were determined in the vascular cambium samples, UG3Y, UG6Y, UG9Y, and UG11Y (Fig. 1). Significant differences in wood chemical composition of *E. urograndis* at different ages were observed. The cellulose content in the vascular cambium sample of UG11Y was different from that in other samples; however, only a little difference in the cellulose content was observed among the vascular cambium samples of UG3Y, UG6Y, and UG9Y. The hemicellulose content was the least in the UG6Y sample, and the hemicellulose content in the UG9Y sample was similar to that in the UG3Y and UG11Y samples. The lignin content in the UG3Y and UG6Y samples was higher than that in the UG9Y and UG11Y samples. The UG9Y sample had low lignin content; however, the contents of cellulose and hemicellulose were high. These results provide a basis for selective processing according to the chemical characteristics of wood.

### Sequencing of *E. urograndis* transcriptome by using the pacbio and illumina platforms

To comprehensively characterize the gene expression dynamics and to identify key genes that regulate the primary and secondary growth of *E. urograndis*, the transcriptomes of *E. urograndis* at four different ages (3, 6, 9, and 11 years) were generated by using the de novo transcript assemblies with paired-end Illumina RNA-seq reads. The full-length transcript analysis was performed using PacBio SMRT sequencing. After quality checks and filtering, 276.54 million 150 bp-paired-end and 82.69 Gb clean data were generated, with the average Q30 and GC percentages of each library being 93.87% and 51.74%, respectively (Table S1). In the SMRT sequencing (Table 1), 485,718 reads of circular consensus (CCS) were generated, which included 426,881 FLNC reads with a mean length of 2,243 bp. The full-length non-chimeric percentage (FLNC%) was 87.89%. Similar full-length non-CCS sequences were clustered into 118,867 consensus isoforms by using SMRTLink software. The sequences had a mean length of 2,147 bp, with 116,624 high-quality consensus isoforms (98.11%) being obtained after polishing. The Illumina RNA-seq data were used to correct the low-quality consensus isoforms, and the corrected consensus isoforms were merged with the high-quality consensus ones (116,624 isoforms) for redundancy analysis. After redundancy removal by using CD-HIT, 59,770 non-redundant transcript isoforms were obtained. The transcript length ranged from 55 to 12,204 bp, with a mean length of 2,322.84 bp and GC content of 46.70%.

**Table 1:** Full-length sequence data statistics of *E. urograndis* at different ages

Parameters	*E. urograndis*
Number of CCS	485,718
Mean read length of CCS	2243 bp
Full-length non-chimeric Reads (FLNC)	426,881
FLNC percentage	87.89%
Number of consensus isoforms	118,867
Average consensus isoforms read length	2147 bp
Number of polished high-quality isoforms	116,624
Percent of polished high-quality isoforms (%)	98.11%
Numer of non-redundant transcript isoforms	59,770
Mean length of non-redundant transcript isoforms	2322.84 bp
GC content of non-redundant transcript isoforms	46.70%

### Structural analysis

TransDecoder software was used to predict the coding region sequence (CDS) of the transcripts and their corresponding amino acid sequence. A total of 57,455 CDSs were obtained, including 45,242 complete CDSs. The predicted length distribution of the com-plete-CDS-encoded protein is displayed in the Figure S1. The highest number of CDSs was observed between 100 and 200 bp, followed by 200 bp and 300 bp, and 300 bp and 400 bp. A total of 1975 and 47 CDSs were less than 1000 bp and 2000 bp, respectively, in length. Moreover, 7102 CDSs were less than 100 bp in length, with a proportion of 12.36%.

The structure of the transcript sequences without redundancy was analyzed and 4,126 AS events, 1240 lncRNAs, and 5680 TFs were obtained in the vascular cambium samples of *E. urograndis* at different ages.

AS events are crucial transcriptional regulatory mechanisms that lead to the structural and functional polymorphisms of transcripts and proteins and play a crucial role in plant growth and development [33]. Among 4126 AS events, 10 were closely related to lignin synthesis, with 14 transcripts being enriched in the phenylpropanoid biosynthesis (ko00940) pathway. Moreover, 34 *CesA*, 38 *SuSy*, and 4 *PGM* (phosphoglucomutase) transcripts were closely related to cellulose biosynthesis, with 30, 25, and 2 AS events, respectively (Table S2).

The venn diagram displays the identification results of non-coding transcripts by using the CPC, CNCI, Pfam, and CPAT analysis methods, with the intersection results (1240) being used for subsequent lncRNA analysis (Table S3). The lncRNA length varied from 200 to 5835 bp, with a mean length of 866.24 bp, which was much shorter than those of 59,770 non-redundant transcript isoforms (2322.84 bp). Moreover, among the 1240 lncRNAs, 371 transcripts had annotation information, and 994 lncRNAs predicted the target transcripts. Among the 994 lncRNAs, 85 predicted 43 target transcripts associated with lignin biosynthesis, 7 predicted 7 target transcripts of *CSL* genes related to hemicellulose biosynthesis, and 129 predicted 65 target transcripts related to cellulose biosynthesis. In addition, a number of genes involved in cell division and differentiation were predicted, including 15 lncRNA that predicted 14 target transcripts of *Expansin-like* genes, 163 lncRNA that predicted 52 target transcripts of *Kenesin-like* genes, 43 lncRNA that predicted 14 target transcripts of *Cyclin-like* genes, and 35 lncRNA that predicted 12 target transcripts of *Histone-like* genes.(Table S4).

A total of 5680 TFs were predicted using ITAK software and classified into 205 types of TFs (Table S5). The core TFs regulating cellulose, hemicellulose, and lignin biosynthesis were *MYB* and *NAC* that contained 108 and 158 transcripts, respectively. These results were combined with those of AS events and 9 transcripts encoding MYB with 5 AS events, and 13 *NAC* transcripts with 9 AS events were identified (Table S2). Moreover, combined with the lncRNA prediction analysis, 52 *NAC* and 34 *MYB* transcripts were predicted by 98 and 61 lncRNAs, respectively.

### Functional annotation

Functional annotation was performed on the 59,770 transcripts, with 57,752 (96.62%) transcripts successfully annotated across the eight protein databases. Among these, 57,703 (96.54%), 44,005 (73.62%), 21,866 (36.58%), 35,074 (58.68%), 23,012 (38.50%), 45,555 (76.22%), 41,030 (68.65%), and 54,961 (91.95%) transcripts were annotated using the NR, GO, KEGG, KOG, COG, Pfam, SwissProt, and eggNOG databases, respectively. Furthermore, similarity analysis between *E. urograndis* transcripts and NR protein databases indicated that *E. urograndis* transcripts matched significantly with homologous genes from *E. grandis* (55,283, 95.81%; Figure S2). In the GO enrichment analysis (Figure S3), the sub-categories of binding (GO:0005488, 23,238 transcripts, 52.81%) in the molecular function (MF) category, cell (GO:0005623, 22,593 transcripts, 51.34%) in the cellular component (CC) category, and the cellular process (GO:0009987, 21,975 transcripts, 49.94%) in the biological process (BP) category were the most prominently represented. The KOG annotation results (Figure S4) revealed that the largest number of unigenes was clustered into the functional category of “General function prediction only” (6843 transcripts, 19.51%), followed by “Signal transduction mechanisms” (4434 transcripts, 12.64%), and “Posttranslational modification, protein turnover, chaperones” (4427 transcripts, 12.62%). Approximately 628 (1.79%) transcripts were assigned to the cluster of “cell wall/membrane/envelope biogenesis”.

**Fig. 2:**
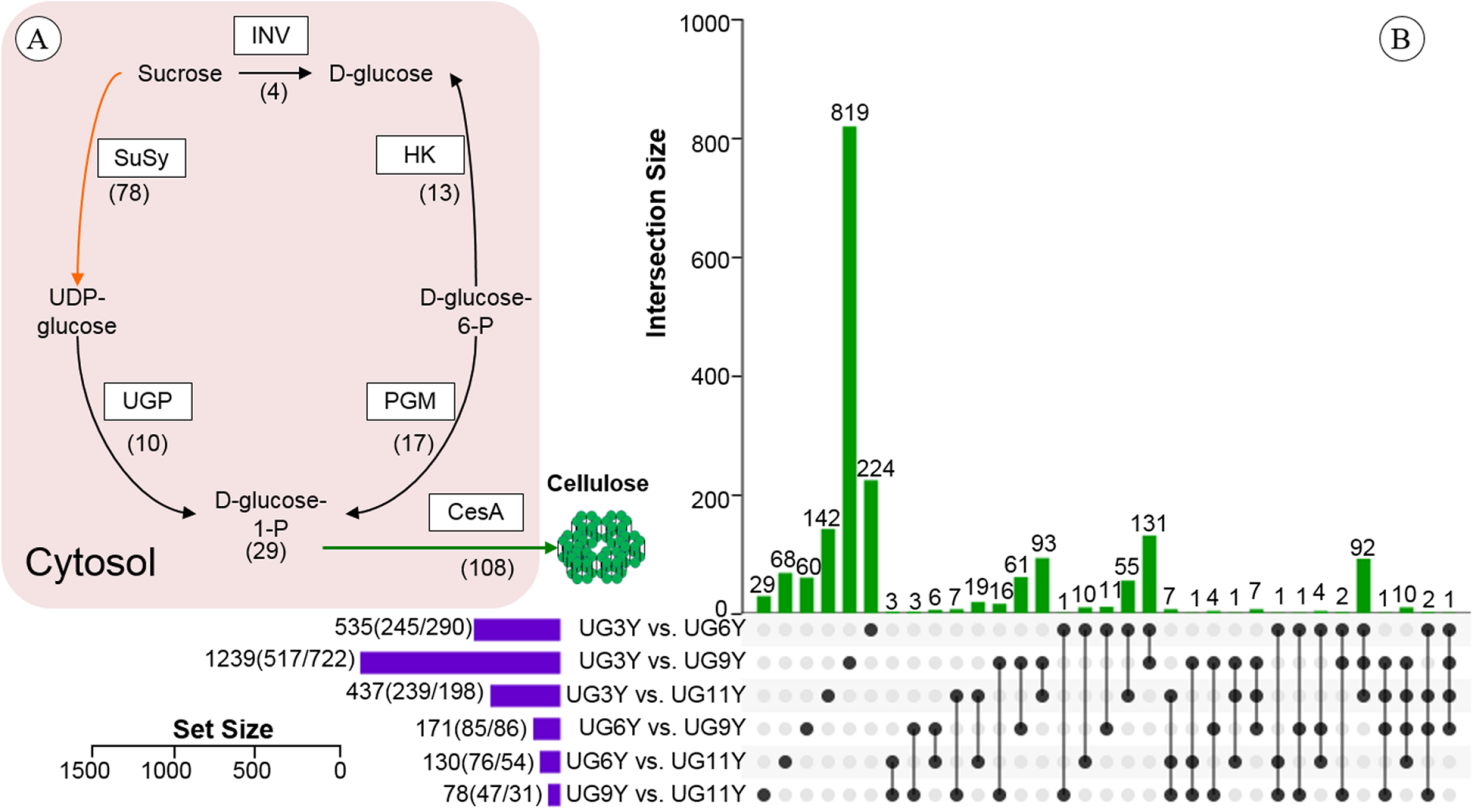
**A** The transcripts encoding cellulose biosynthesis enzymes identified in the vascular cambium of *E. urograndis*. The icons represent the enzymes and the number in parentheses below icons indicate the all transcript number encoding the enzyme. **B** UpSet of DEGs between pairwise comparison by any two samples in the vascular cambium at different ages of *E. urograndis*. The horizontal bar chart on the left shows the number of DEGs in various comparable groups (up-regulated/down-regulated), the lines between points represent the intersection of various comparable groups, and the vertical bar chart respectively represents the corresponding number of intersection comparable groups

On the basis of KEGG pathway assignment, 126 pathways (Table S6) were assigned, with the majority of these transcripts involved in the pathways of protein processing in the endoplasmic reticulum (ko04141, 818 transcripts, 3.74%), carbon metabolism (ko01200, 766 transcripts, 3.50%), and amino acid biosynthesis (ko01230, 706 transcripts, 3.23%). Moreover, 167 transcripts were assigned to the phenylpropanoid biosynthesis (ko00940) pathway (Table S7), which is strongly associated with lignin biosynthesis. The transcripts encoding cellulose biosynthesis enzymes were identified and included 78 *Susy*, 108 *CesA*, 4 *INV*, 13 *HK*, 17 *PGM*, and 10 *UGP* transcripts (Fig. 2A).

### Differentially expressed genes in *E. urograndis* at different ages

To identify DEGs, six paired comparisons were performed: UG3Y vs. UG6Y, UG3Y vs. UG9Y, UG3Y vs. UG11Y, UG6Y vs. UG9Y, UG6Y vs. UG11Y, and UG9Y vs. UG11Y. A total of 1892 DEGs were identified and an UpSet visualization [34] was used to diplay the relationships between data sets (Fig. 2B). In UG3Y vs. UG9Y, the number of DEGs was up to 1239 (517 upregulated and 722 downregulated), whereas the number of DEGs was only 78 (47 upregulated and 31 downregulated) in UG9Y vs. UG11Y. Therefore, a greater difference in transcription level was the largest in UG3Y vs. UG9Y, followed by UG3Y vs. UG6Y and UG3Y vs. UG11Y.

GO functional enrichment analysis was performed for DEGs in each comparison group (Table S8). The results revealed 384 DEGs enriched in 1514 GO terms in UG3Y vs. UG6Y, 928 DEGs were enriched in 2250 GO terms in UG3Y vs. UG9Y, and 316 DEGs were enriched in 1421 GO terms in UG3Y vs. UG11Y. In UG6Y vs. UG9Y, 132 DEGs were enriched in 1018 GO terms, 94 DEGs were enriched in 870 GO terms in UG6Y vs. UG11Y, and 61 DEGs were enriched in 710 GO terms in UG6Y vs. UG11Y. The GO terms of oxidation reduction process (GO:0055114) and regulation of transcription, DNA-templated transcription (GO:0006355) were the most frequent in the BP category. The GO terms of integral component of membrane (GO:0016021) and cytosol (GO:0005829) were the most frequent in the CC category. The GO terms of structural constituent of ribosome (GO:0003735) and catalytic activity (GO:0003824) were the most frequent in the MF category.

The KEGG pathway enrichment analysis was performed for DEGs in each comparison group (Table S9). The results revealed 88 DEGs enriched in 50 KEGG pathways in UG3Y vs. UG6Y, 207 DEGs enriched in 77 KEGG pathways were identified in UG3Y vs. UG9Y, and a total of 98 DEGs enriched in 43 KEGG pathways were identified in UG3Y vs. UG11Y. Other three comparison groups, UG6Y vs. UG9Y, UG6Y vs. UG11Y, and UG9Y vs. UG11Y, 24, 31 and 18 DEGs were enriched 18, 33 and 19 KEGG pathway, respectively. Among those pathways, the starch and sucrose metabolism (ko00500) and phenylpropanoid biosynthesis (ko00940) were most significant, especially in the comparison groups of UG3Y vs. UG6Y and UG3Y vs. UG9Y.

### Expression patterns of DEGs related to cell division and differentiation

**Fig. 3:**
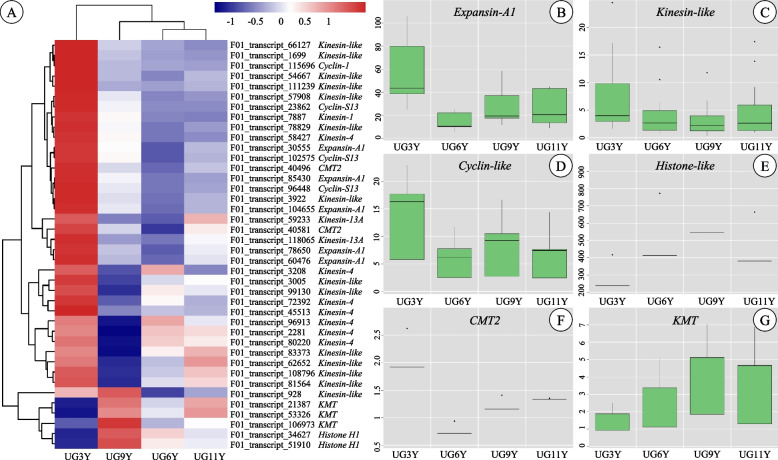
Expression profile of the DEGs involved in cell division and differentiation among four ages of *E. urograndis*. **A** Heatmap shows expression of DEGs associated with cell division and differentiation. **B**-**G** Boxplots show expression of all transcripts encoding Expansin-A1, Kinesin-like, Cyclin-like, Histone-like, CMT2 and KMT, respectively

Cell division and differentiation occur in the cambium zone and are crucial for vascular tissue growth, leading to stem thickening [35]. The regulation of cell division and differentiation in vascular cambium is a dynamic and highly regulated process. In this study, five DEGs (F01_transcript_60476, F01_transcript_78650, F01_transcript_104655, F01_transcript_30555, and F01_transcript_85430) encoding expansin-A1 were all significantly upregulated in UG3Y (Fig. 3A and B). Similar to *expansin-A1* transcripts, the relative expression levels of 42 *kinesin-like* and 4 *cyclin-like* transcripts were also higher in UG3Y than in other ages of *E. urograndis* (Fig. 3A, C and D). This may be because expansin, kinesin, and cyclin are crucial for plant growth and development as they promote cell division and differentiation. By contrast, the expression of *histone-like* was inhibited to some extent in UG3Y. The expression levels of the other two *histone-H1* transcripts (F01_transcript_34627, F01_transcript_51910) were higher in the vascular cambium of *E. urograndis* (FPKM > 200) at four different ages (Fig. 3A and E). This may be attributable to the important role of histone-H1 in repairing DNA and protein damage and maintaining genome stability [36].

The DNA methylation levels changed with tree age [37]. This study analyzed the differential expression of DNA methylation-related genes in the vascular cambium of trees at four different ages. The two transcripts of *CMT2* were downregulated in UG6Y, whereas three *KMT* transcripts were downregulated in UG3Y and stably expressed in UG9Y and UG11Y (Fig. 3A, F and G).

### Expression patterns of DEGs related to secondary cell wall biosynthesis

**Fig. 4:**
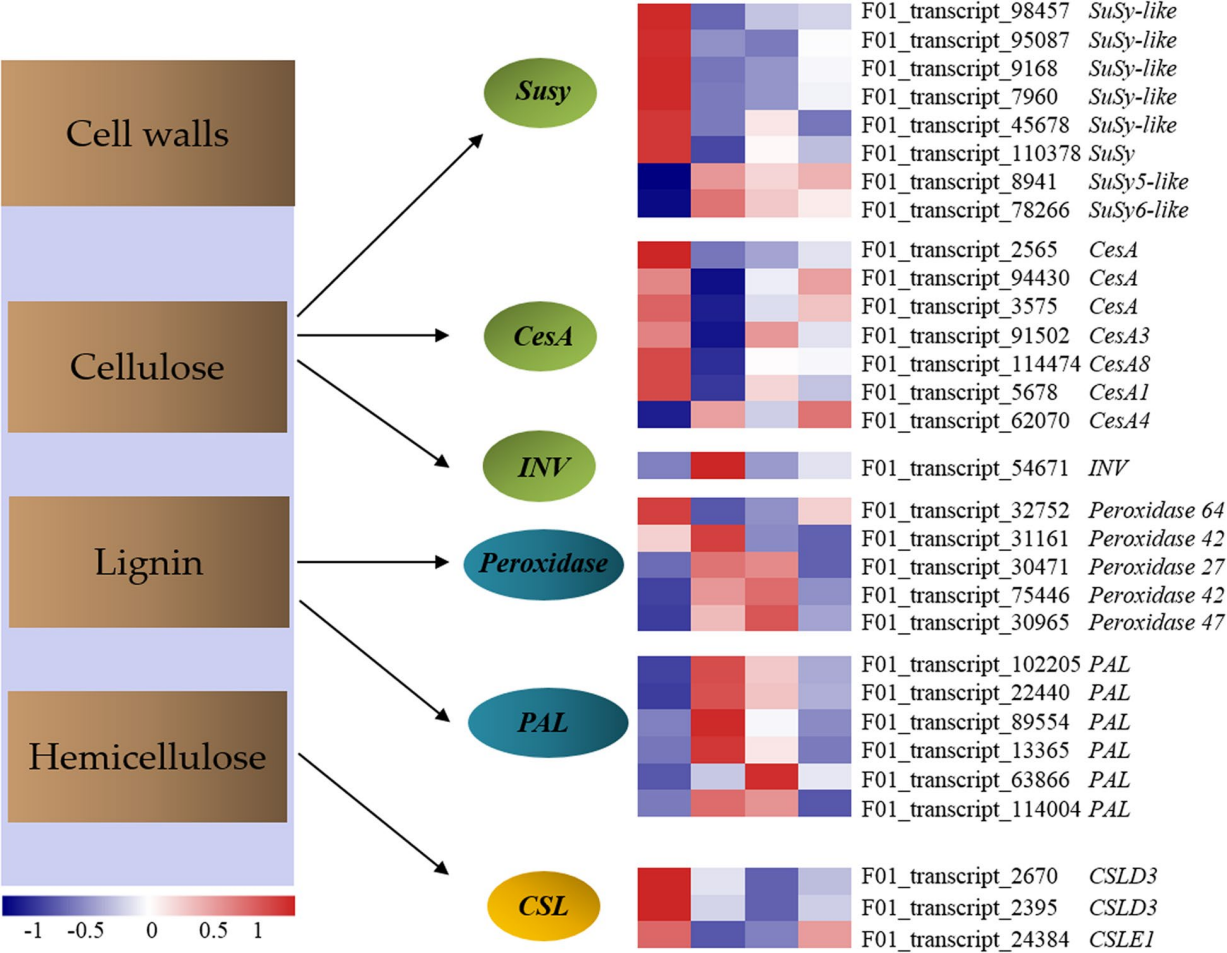
Expression profile of the DEGs involved in cell wall biosynthesis among four ages of *E. urograndis*

Secondary cell wall biosynthesis is associated with gene expression in the vascular cambium, with the process playing a critical role in wood formation. Vascular cambium differentiation and development play a decisive role in secondary growth, thereby determining the yield, structure, and properties of wood. More than 90% of the total material content in the cell wall was made up of cellulose, hemicellulose, and lignin. To identify key genes involved in the changes of cellulose, hemicellulose, and lignin contents in the *E. urograndis* vascular cambium of different ages, the DEGs related to cellulose biosynthesis from sucrose, *CSL* family, and lignin biosynthesis from phenylalanine were analyzed (Fig. 4). The cellulose biosynthesis pathway from sucrose had 15 DEGs, including 6 *CesA*, 8 *Susy*, and 1 *INV* transcripts. All six *CesA* transcripts were downregulated in UG9Y, and F01_transcript_94430 encoding *CesA4* was significantly upregulated in UG11Y than UG9Y in UG9Y vs. UG11Y. *CesA4* was upregulated in tissues rich in the rapidly dividing cells undergoing primary wall synthesis in *E. urograndis* [38, 39], which was consistent with the growth characteristics between UG9Y and UG11Y (DBH growth or primary growth was more). The expression of F01_transcript_9168 encoding Susy was the highest in UG3Y. The other two *Susy* transcripts (F01_transcript_8941 and F01_transcript_78266) and one *INV* transcript (F01_transcript_54671) were upregulated in UG9Y; however, their expression levels were low (FPKM < 5). Moreover, sucrose transport proteins (STPs) are proton-coupled symporters responsible for uptake of glucose from the apoplast into plant cells [40]. Two transcripts (F01_transcript_105532_and F01_transcript_115148) that encode STP were considerably downregulated in UG3Y and UG11Y and significantly upregulated in UG6Y. Therefore, it can be concluded that the vascular cambium of *E. urograndis*, which is 6_years_old, is quite active, whereas the 11_years_old is substantially less active.

There were eleven DEGs (5 *Peroxidase* amd 6 *PAL*) were enriched in the phenylpropanoid biosynthesispathway (ko00940) (Fig. 4). Among them, the expression of F01_transcript_32752 encoding peroxidase 64-like was high in UG3Y and UG11Y. Three DEGs (F01_transcript_75446 encoding peroxidase 42-like, F01_transcript_30965 encoding peroxidase 47-like, and F01_transcript_63866 encoding PAL) were highly expressed in UG6Y, which may play a positive role in lignin biosynthesis and may be associated with the higher lignin content in UG6Y than in other three samples.

There were four DEGs were identified in the *CSL* family, with two* CslD3* and one *CslE1* transcripts having the same expression patterns (Fig. 4). The expression levels of these transcripts were relatively higher in UG3Y than in other samples. The expression pattern was similar to the dynamic change of hemicellulose content in *E. urograndis* samples of different ages, which inferred that the three transcripts might promote hemicellulose biosynthesis in the vascular cambium of *E. urograndis*.

### Expression patterns of phytohormone-related DEGs

**Fig. 5:**
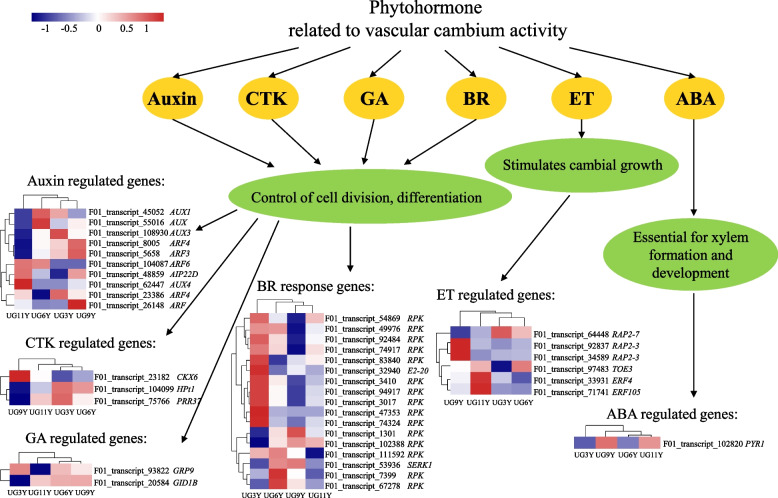
Expression profile of the DEGs involved in phytohormone signaling among the four ages of *E. urograndis*. Notes: CTK, cytokinin; GA, gibberellin; BR, brassinosteroid; ET, ethylene, and ABA, abscisic acid

Various phytohormone signals and their interactions play an important role in vascular differentiation, with phytohormones regulating wood formation through a complex regulatory network [41]. Auxin is a crucial phytohormone that regulates vascular cambium development and the formation of plant secondary structures. The gradient distribution and polar transport of auxin play a crucial role in the differentiation of xylem cell [15]. Among the 10 DEGs related to the response, transduction, and induction of auxin signal (Fig. 5), the expression levels of three transcripts (F01_transcript_5658, F01_transcript_8005 and F01_transcript_26148) encoding auxin response protein (ARF) were higher in UG9Y. The relative expression level of F01_transcript_48859 encoding auxin–induced protein 22D (AIP22D) increased with tree age, while three transcripts (F01_transcript_45052, F01_transcript_55016 and F01_transcript_108930) encoding auxin transporter protein (AUX) downregulated in UG11Y. Cytokinin is a major plant hormone that promotes cell division in various meristems [42]. In this study, F01_transcript_23182 encoding cytokinin dehydrogenase 6 (CKX6) had a high relative expression level in UG9Y, whereas F01_transcript_104099 encoding histidine-containing phosphotransfer protein 1 (HPt1) and F01_transcript_75766 encoding two-component response regulator (PRR37) were highly expressed in UG3Y (Fig. 5). Ramachandran et al. [43] and Campbell et al. [44] demostrated that an essential role of abscisic acid (ABA) in xylem formation and development. Among the abscisic-acid-related DEGs, F01_transcript_102820 encoding abscisic acid receptor (PYR1) was relatively highly expressed in UG9Y (Fig. 5). Gibberellins induce cell differentiation and lignification in the vascular cambium [45]. In this study, the expression level of the *GID1B* transcript (F01_transcript_93822) was relatively lower in UG3Y than in other samples, and the relative expression level of the GRP9 transcript (F01_transcript_20584) was upregulated in UG3Y (Fig. 5). Brassinosteroids (BRs) are a key promoting signal for procambial division during primary growth [7]. Among 17 BR-related DEGs (Fig. 5), 10 transcripts encoding LRR receptor-like serine/threonine-protein kinase (RPK) were upregulated in UG3Y. The expression of F01_transcript_32940 encoding ubiquitin-conjugating enzyme (E2-20) was higher in UG3Y than in other samples, and F01_transcript_53936 encoding somatic embryogenesis receptor kinase 1 (SERK1) was relatively highly expressed in UG6Y and UG9Y. When applied exogenously, the gaseous plant hormone ethylene stimulates cambial growth and induces typical features of reaction wood (such as G-layer formation) [6]. There were six DEGs related to ethylene biosynthesis were identified (Fig. 5), with two transcripts (F01_transcript_34589 and F01_transcript_92837) encoding RAP2–3 being upregulated in UG9Y. The transcripts encoding ERF4 and ERF105 (F01_transcript_33931 and F01_transcript_71741, respectively) were highly expressed in UG11Y; F01_transcript_64448 encoding RAP2–7 was highly expressed in UG3Y, and F01_transcript_97483 encoding TOE3 was upregulated in UG6Y.

### Expression patterns of TF-related DEGs

**Fig. 6:**
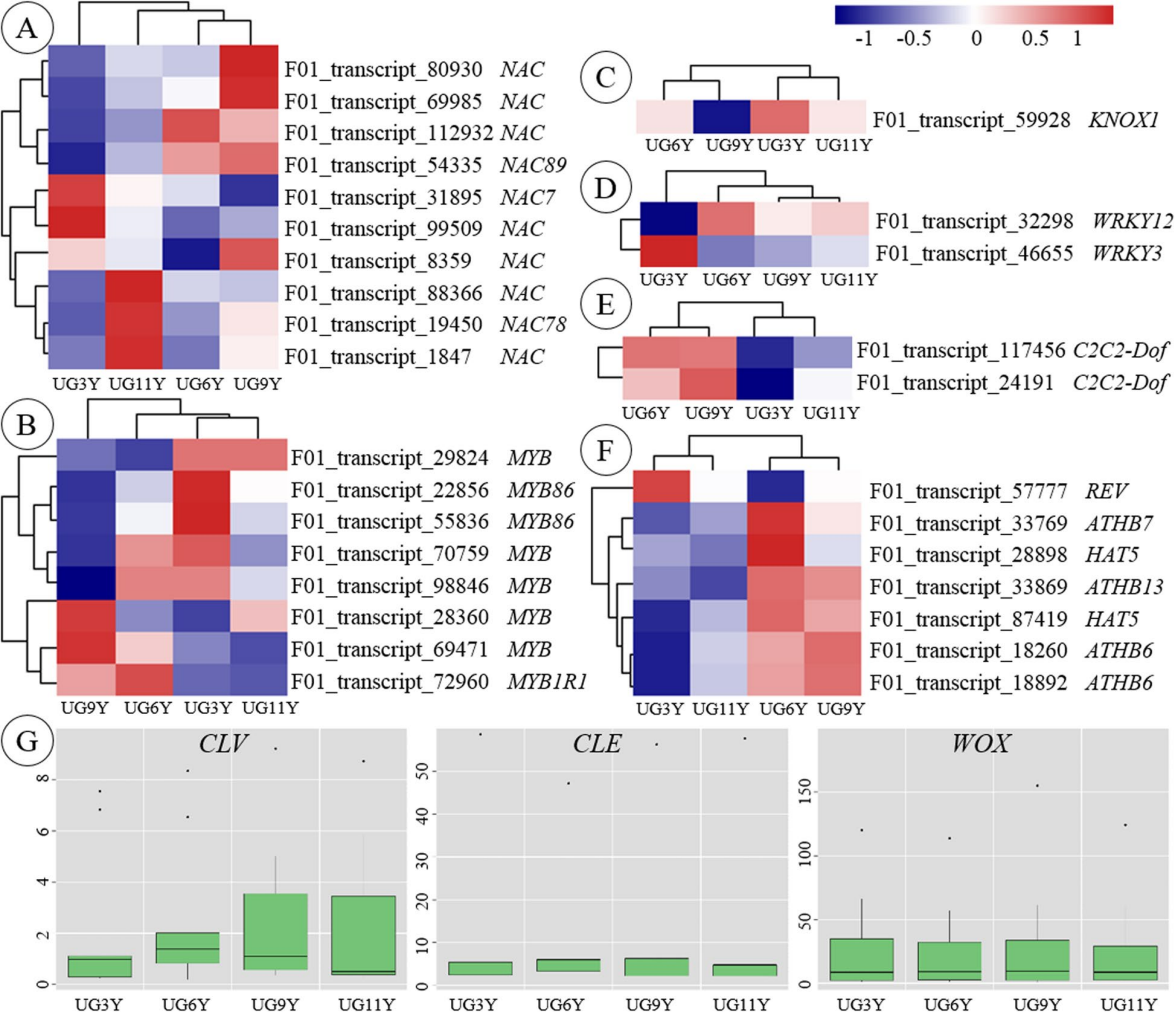
Expression profile of the differentially expressed TFs among the four ages of *E. urograndis*. Heatmap shows expression of differentially expressed TFs of **A** *NAC*, **B** *MYB*, **C** *KONX*, **D** *WRKY*, **E** *C2C2-Dof*, and **F** *HD-ZIP III*. **G** Boxplots show expression of transcripts encoding CLV, CLE and WOX

TFs regulate plant development, secondary metabolism, and biological and abiotic stress [46]. After the analysis of core TFs regulating cellulose, hemicellulose, and lignin biosynthesis, 10 *NAC* and 8 *MYB* transcripts were identified as DEGs (Fig. 6A and B). Of these, two *NAC* transcripts (F01_transcript_31895 and F01_transcript_99509) were upregulated in UG3Y, and five MYB transcripts were upregulated in UG3Y. In this study, KNOX (KNOTTED HOMEOBOX) TFs were analyzed because of their regulatory role in the vascular cambium [47]. In UG3Y, F01_transcript_59928 encoding KNOX1 was highly expressed (Fig. 6C). In addition, *WRKY* and *C2C2-dof* are involved in plant growth and development and abiotic-stress-related processes. The two DEGs (F01_transcript_117456 and F01_transcript_24191) encoding WRKY were highly expressed in UG6Y and UG9Y (Fig. 6D). Among the two DEGs encoding C2C2-dof, F01_transcript_32298 was upregulated in UG6Y, and F01_transcript_46655 was highly expressed in UG3Y (Fig. 6E).

In *Populus*, *HD-ZIP III* is associated with auxin signaling pathways and regulates asymmetric auxin distribution in multiple developmental processes [26]. Seven DEGs encoding HD-ZIP III were identified in this study (Fig. 6F). Of these, F01_transcript_57777 was downregulated in UG6Y, whereas the other six transcripts were upregulated in UG6Y or UG9Y. As members of the *HD-ZIP III* subfamily, four *ATHB6/7/13* transcripts (F01_transcript_18260, F01_transcript_18892, F01_transcript_33769, and F01_transcript_33869) and two *HAT5* transcripts (F01_transcript_28898 and F01_transcript_87419) were upregulated in UG6Y and UG9Y. By contrast, *REVOLUTA* (*REV*) transcript (F01_transcript_57777), another member of the *HD-ZIP III* subfamily, had a higher expression level in UG3Y than in other samples.

Plant stem cells in the vascular cambium balance cell division and differentiation, thereby constantly forming new tissues. The main TFs affecting plant stem cell viability include *CLV*, *CLE*, and *WOX*. In this study, the expression levels of six *CLV*, three *CLE*, and eight *WOX* transcripts were similar across four different ages of *E. urograndis* (Fig. 6G), suggesting their important role in maintaining the sustainable growth of *E. urograndis*.

### Validation of candidate DEGs involved in vascular cambium at different ages

To ensure the transcriptome data accuracy, 18 candidate genes, with relatively high expression levels and read counts (FPKM ≥ 10 and read counts ≥ 20), involved in vascular cambium of *E. urograndis* at different ages were selected for real-time quantitative reverse transcription polymerase chain reaction (RT-qPCR). The relative expression profiles of 18 candidate genes by RT-qPCR were consistent with relative expression trends in the transcriptome (Figure S5A-R). Moreover, a highly significant correlation (*R*^2^ = 0.9034) was obtained for RT-qPCR and transcriptome data (Figure S5), which suggested that the transcriptome data were highly reliable and reflected the expression levels of all genes in the study.

## Discussion

Plant secondary growth, which is the basis of wood formation, consists of the production of secondary xylem from meristematic cambium cells embedded in vascular tissue [48]. The secondary growth of vascular tissue in the perennial tree stems is the biological basis of wood growth, with the formation of vascular tissue being dependent on the continuous proliferation and differentiation of vascular cambium cells [49]. Vascular cambium activity determines the yield, structure, and property of timber, thereby playing a key role in wood formation and wood gene regulation [50]. Therefore, the understanding of genetic mechanisms that regulate vascular cambium at different ages from the perspective of transcriptomics can provide a theoretical basis for analyzing the regulatory mechanism underlying vascular cambium activity in woody plants. Moreover, it can provide resources for genetic-engineering-based breeding to further improve wood yield and quality in the future.

Our study results indicated that the cellulose, hemicellulose, and lignin contents were significantly different among the vascular cambium of *E. urograndis* at different ages. This may be attributable to the gene expression in the vascular cambium. Differences in the expression of genes related to the regulation of cell division and differentiation, cell wall biosynthesis, and phytohormones were observed among the *E. urograndis* vascular cambium at different ages. Therefore, this study combined the Pacbio SMRT and Illumina sequencing to analyze transcriptional regulation in the vascular cambium of the dominant hybrid species *E. urograndis* at four different ages. The candidate genes in vascular cambium responding to change in tree age of *E. urograndis* were screened by analyzing the DEGs, thereby providing a theoretical basis for analyzing the regulatory mechanism of wood formation.

Full-length transcript analysis was performed for the *E. urograndis* vascular cambium at four ages by using Pacbio SMRT sequencing, leading to the generation of 40.37 Gb of clean data. Through transcriptome sequencing of 12 samples, 82,69 Gb of clean data were obtained, with Q30 reaching over 85%. In the full-length transcriptome, 426,881 FLNC reads with a mean length of 2,243 bp were obtained. A total of 116,624 high-quality con-sensus isoforms were obtained from 118,867 consensus isoforms after polishing. After redundancy correction, 59,770 non-redundant transcripts with a mean length of 2,322.84 bp and GC content of 46.70% were obtained. The structural analysis of the 59,770 non-redundant transcripts led to the prediction of 45,242 complete CDSs, 4126 AS events, 1240 lncRNAs, and 5680 TFs. Furthermore, 57,752 transcripts were annotated across the eight protein databases.

**Fig. 7:**
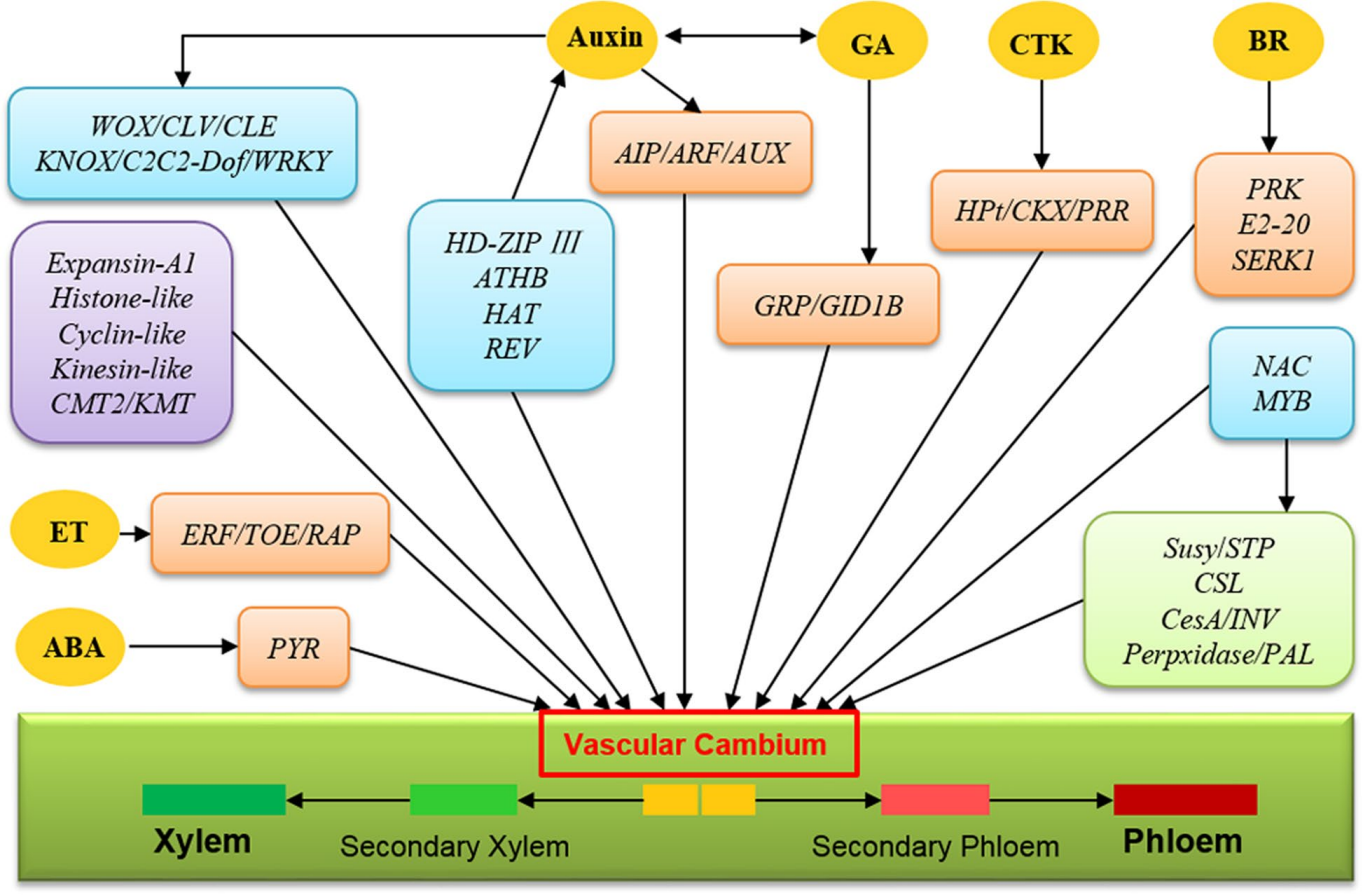
Transcriptional and hormonal regulation on vascular cambium of *E. urograndis* responding to tree age changes. Blue boxes indicate transcription factors (TFs), purple box indicates DEGs related to cell division and differentiation, light green box indicates DEGs related to secondary cell wall biosynthesis, and orange boxes indicate phytohormone-related DEGs. Phytohormones include auxin, cytokinin (CTK), brassinosteroid (BR), gibberellin (GA), ethylene (ET), and abscisic acid (ABA)

In this study, DEGs were analyzed to determine significant changes of genes in the vascular cambium that responded to the tree age changes of *E. urograndis*. A total of 1892 DEGs were screened, with the largest number identified in UG3Y vs. UG9Y, followed by UG3Y vs. UG6Y, and UG3Y vs. UG11Y. The lowest number of DEGs (78) were identified in UG9Y vs. UG11Y, thereby indicating that the gene expression patterns were similar in the vascular cambium aged 9 and 11 years. This was consistent with the changes in the lignin and hemicellulose contents. The KEGG enrichment analysis of DEGs revealed DEGs enriched in the starch and sucrose metabolism (ko00500) pathway in the four comparison groups. Three comparable groups had DEGs enriched in the phenylpropanoid biosynthesis pathway (ko00940). These pathways are closely associated with cellulose and lignin biosynthesis [51, 52], indicating that DEGs related to cellulose and lignin biosynthesis in *E. urograndis* vascular cambium at different ages may be related to the changes in cellulose and lignin contents (Fig. 7). In UG3Y, six *CesA* and six *Susy* DEGs were upregulated. This may be related to the higher content of cellulose in the vascular cambium of 3-year-old *E. urograndis* than in other samples. In the phenylpropanoid biosynthesis pathway, two DEGs of *Peroxidase* were upregulated in UG6Y, which is consistent with the highest lignin content in 6-year-old *E. urograndis*. Because core genes mediate hemicellulose biosynthesis, three DEGs of *CSL* were upregulated in UG3Y, which is inconsistent with the hemicellulose content changes in *E. urograndis* vascular cambium at different ages. Therefore, other key regulators may be involved in cellulose, lignin, and hemicellulose biosynthesis (Fig. 7). *NAC* TFs are master regulators of the secondary cell wall transcriptional regulatory network and play a positive regulatory role in secondary cell wall formation (Fig. 7) [53, 54]. For example, vascular-related *NAC domain 6/7* (*VND6* and *VND7*) are key regulatory factors of secondary and primary xylem formation [55]. In this study, F01_transcript_31895 encoding NAC domain 7 was upregulated in UG3Y, which is consistent with the higher cellulose content in 3-year-old *E. urograndis* than in other samples. *MYB* TFs are indispensable for secondary cell wall biosynthesis [56] and regulate its formation by promoting or inhibiting lignin, cellulose, and hemicellulose biosynthesis (Fig. 7) [57]. F01_transcript_2469 encoding MYB3R-1 was upregulated in UG3Y, which may be related to cell cycle control [58]. F01_transcript_29824 encoding MYB46 promotes cellulose, lignin, and hemicellulose biosynthesis in *Arabidopsis* [59] and poplar [60]. The expression level of this transcript was high in UG3Y and UG11Y, which is consistent with the results of cellulose, lignin, and hemicellulose contents in different ages of *E. urograndis*. Studies have also demonstrated that some *NAC* and *MYB* members repress the lignin biosynthesis pathway [23, 61, 62]. In the vascular cambium at different ages of *E. urograndis*, F01_transcript_1847 and F01_transcript_19450 encoding NAC domain 86 were upregulated in UG11Y, which was opposite to the results of cellulose and lignin contents in different ages of *E. urograndis*. The two transcripts may have similar biological functions as *XND1* in *Arabidopsis* [53, 61]. In addition, F01_transcript_101095 and F01_transcript_91734 encoding MYB308 are negative regulators of lignin biosynthesis [57] and were upregulated in UG9Y and UG11Y, which is opposite to the results of *E. urograndis* at different ages. Therefore, the different expression profiles of NAC and MYB TFs may be attributed to their various biological functions.

The vascular cambium of trees is a crucial lateral meristem [63]. The maintenance of cell division and differentiation in the wood vascular cambium is essential for continuous tree thickening [64], thereby determining the growth rate and tree yield [65]. Secondary growth is initiated by the assembly of the vascular cambium as a continuous meristematic layer. The genes involved in vascular cambium regulation are similar to those involved in the regulation of shoot and root apical meristem [66]. Studies have demonstrated that the TDIF/CLE41/CLE44-TDR/PXY-WOX4 and HD-ZIP/auxin/PIN (pin-formed proteins)/ARF5 signaling pathways maintain and promote shoot and root apical meristem formation in *Arabidopsis* and *Populus* (Fig. 7) [26, 64, 67]. In this study, one DEG of *PIN*, 5 DEGs of *expansin-A1*, 27 DEGs of *kinesin-like*, and 4 DEGs of *cyclin-like* were upregulated in 3-year-old *E. urograndis*. The four DEGs encoding ATHB6/7/13 and two DEGs encoding HAT5 were highly expressed in UG6Y and UG9Y; however, F01_transcript_57777 encoding REV was upregulated in UG3Y. These results are consistent with those of Kim et al. [68] and Prigge et al. [69], thereby suggesting that *REV* and *ATHB15* have opposite effects on vascular pattern regulation. The transcripts of *WOX* and *CLV* are key regulators of WUSCHEL/CLAVATA (WOX/CLV) that maintain cell division and differentiation in the vascular cambium, and these transcripts had similar expression levels in the vascular cambium of *E. urograndis* at four different ages. Moreover, five DEGs of *ARF* had relatively higher and more stable expression levels in UG3Y, UG6Y, and UG9Y than in other samples. AGL14, which transmits endogenous stress-related signal (ESS) signals to the WOX/CLV network, was upregulated in UG6Y. Therefore, the *E. urograndis* vascular cambium at four ages exhibited an optimal ability of cell division and differentiation, and young age was associated with a greater ability of cell division and differentiation in the vascular cambium.

Extensive studies in *Arabidopsis* and trees have demonstrated that phytohormones are involved in regulating the growth and development of higher plants (Fig. 7) [7, 70]. In this study, three auxin transporter protein transcripts (F01_transcript_108930, F01_transcript_45052, and F01_transcript_55016) and two auxin response factor transcripts (F01_transcript_8005 and F01_transcript_5658) were downregulated in UG11Y, which is consistent the changes in cellulose and lignin contents in different ages of *E. urograndis*. F01_transcript_48859 encoding auxin-induced protein had a higher expression level in UG9Y and UG11Y than in other samples, which is consistent with the changes in the hemicellulose content at different ages of *E. urograndis*. The expression profiles of these auxin-related genes were consistent with the function of IAA, which promotes xylem and phloem differentiation in poplar [71] and *Eucommia ulmoides* [72]. Ben-Targem et al. [24] discussed the pivotal phytohormone cross-talk between IAA and GA in vascular cambium growth. In this study, the expression level of F01_transcript_93822 encoding GRP9 decreased gradually with *E. urograndis* growth, which is consistent with the changes in the expression level of F01_transcript_48859 encoding auxin-induced protein (AIP). Immanen et al. [73] and Rahimi et al. [48] reported that cytokinins are major rate-limiting phyto-hormonal regulators of cambial development in *Populus* and *Arabidopsis*. F01_transcript_23182 encoding cytokinin dehydrogenase 6 (CKX6) had higher expression levels in UG9Y and UG11Y, and the *E. urograndis* growth rate reduces with the dynamic changes of height and DBH. Moreover, the role of ethylene as endogenous stimulator of vascular cambial activity in *Arabidopsis* [74] and poplar [75] was elucidated. The expression trends of two DEGs (F01_transcript_33931 and F01_transcript_71741) and F01_transcript_64448 encoding ERF were consistent with the changes in hemicellulose and lignin contents, respectively, with growth.

TFs play crucial regulatory roles in regulating plant development, secondary metabolism, biotic and abiotic stresses, as well as other biological processes [5, 76, 77]. In addition to *MYB*, *NAC*, *HD-Zip III*, and *WOX*, other TFs, such as *KNOX*, *WRKY*, and *C2C2-dof*, influence cell division and differentiation in the vascular cambium (Fig. 7). Zhao et al. [78] and Reyes-Rivera et al. [79] reported that *KNOX* TFs that play important roles in vascular cambium activity maintenance and xylem differentiation. In this study, the *KNOX1* transcript (F01_transcript_59928) was upregulated in UG3Y, which is similar to that of *PtKNAT2/6b*, *ARBORKNOX1* (ARK1), and *ARK2* in *Populus* [78]. Studies have suggested that some *WRKY* members negatively regulate secondary cell wall formation in *Populus* [80]. In this study, the *WRKY12* transcript (F01_transcript_32298) was downregulated in UG3Y, thereby indicating that *PtWRKY12* homologous gene *WRKY12* in *E. urograndis* negatively regulates secondary cell wall formation. The C2C2-dof zinc finger family are involved in cellulose and lignin metabolism and secondary cell wall biosynthesis [81, 82]. In the *E. urograndis* vascular cambium, two *C2C2-dof* transcripts were upregulated in UG6Y and UG9Y, which is consistent with the higher cellulose and lignin contents in UG6Y and UG9Y than in other samples. Therefore, *C2C2-dof* transcripts may have a positive impact on the wood formation of *E. urograndis*.

## Conclusions

In this study, RNA-Seq and Pac-Bio Iso-Seq were used for profiling changes in gene expression in the vascular cambium of *E. urograndis* at different ages. A total of 59,770 non-redundant transcripts were identified, and 1892 DEGs were screened across the six comparison groups. Further analysis revealed that these DEGs were related to cell division and differentiation, cell wall biosynthesis, phytohormone, and TFs. The DEGs, such as *Expansin*, *Kinesin*, *Cycline*, *PAL*, *GRP9*, *KNOX*, *C2C2-dof*, and *REV*, with positive effects on growth and development were highly expressed in UG3Y and UG6Y. Some gene family members, such as *NAC*, *MYB*, *HD-ZIP III*, *RPK*, and *RAP* function redundantly and play different regulatory roles in the vascular cambium of *E. urogandis* responding to tree age changes because of their sophisticated transcriptional network. These candidate genes are a potential resource to further study vascular cambium activity and wood formation, especially in fast-growing and adaptable eucalyptus.

## Methods

### Biological material

Tissue samples were harvested from *E. urograndis* clonal trees (DH32–29) of four different ages (3, 6, 9, and 11 years), which were cultivated at the South China Experimental Nursery (longitude 111°38′ E, latitude 21°30′ N, and altitude 90 m above sea level). The tissues from the cambial region in the diameter at breast height (DBH) of trees were collected on September 18, 2020, when the growth was rapid, and the vascular cambium activity was high. The bark of each tree was removed by exposing an area of approximately 15 × 10 cm^2^. The stem-exposed tissue and inner surface of the bark were scraped using a sterile blade, and the samples were immediately frozen in liquid nitrogen and stored at − 80 °C for further study. The detail information of all tree samples are presented in Table S10.

### Wood property analyses

Three wood properties (cellulose, hemicellulose, and lignin contents) of *E. urograndis* samples were measured. A double antibody sandwich enzyme-linked immunosorbent assay (ELISA) was used for the measurement of cellulose, hemicellulose, and lignin contents by using the plant cellulose, hemicellulose, and lignin ELISA Kits (Jining Shiye, Shanghai, China), respectively [39]. The concentrations of cellulose, hemicellulose, and lignin in the samples were determined by comparing the OD values of the samples with those of the standard. The data were examined using analysis of variance and subjected to least significant difference multiple comparisons by using SPSS Statistics 19.0.

### Illumina RNA-seq and PacBio library construction and sequencing

Total RNA was isolated from the 12 vascular cambium samples (three biological replicates from each of the four ages of *E. urograndis*) by using an RNAprep pure PlantKit (TIANGEN Biotech, Beijing, China) with DNase I treatment to remove genomic DNA. The concentration and integrity of the extracted total RNA were evaluated using the Agilent RNA 6000 Nano Kit and the Agilent 2100 Bioanalyzer System (Agilent Technologies, Palo Alto, CA, USA). High-quality RNA (RNA integrity number for all samples was higher than 9.0) was used for sequencing library construction.

The sequencing libraries were generated using the NEBNext Ultra RNA Library Prep Kit for Illumina (NEB). The RNA-seq was performed on the Illumina HiSeq 4000 sequencing system (Illumina, San Diego, CA, USA) based on the paired-end 150 strategy by Biomarker Technologies Co. (Beijing, China).

Total RNA isolated from the *E. urograndis* samples of four different ages was mixed equally and pooled for the PacBio library construction. Full-length cDNA was synthesized using the SMARTer PCR cDNA Synthesis Kit (Clontech Laboratories, California, USA). BluePippin (Sage Science, Massachusetts, USA) was used for size selection of the full-length cDNA and for the construction of differently-sized cDNA libraries. Single-molecular real-time (SMRT) libraries were generated using the PacBio SMRTbell Template Prep Kit 1.0 (PacBio, Menlo Park, CA, USA). Sequencing was performed using the PacBio RS II system (PacBio, Menlo Park, CA, USA).

All raw transcriptome sequences were deposited to the Sequence Read Archive at the National Center for Biotechnology Information (NCBI) with the accession number of SRP425632.

### Bioinformatic analysis of PacBio and illumina data

To obtain high-quality clean reads, raw data were further filtered by using FASTP (0.18.0). Subsequently, clean data were mapped to the reference genome (*E. grandis*: GCF_000612305.1) by using Hisat2 (2.0.3.12). The transcriptome integrity was assessed using Benchmarking Universal Single-Copy Orthologs 3.0.2, with OrthoDBs as the reference database. Gene function was annotated using eight databases, namely NR (NCBI non-redundant protein sequences), Pfam (protein family), KOG/COG/eggNOG (Cluster of Orthologous Groups of proteins), Swiss-Prot, KEGG (Kyoto Encyclopedia of Genes and Genomes), and GO (Gene Ontology). GO and KEGG pathway enrichment analysis of the transcripts was performed using the GOseq R package 3.4.3 and KOBAS software [83, 84].

Raw reads were processed to obtain error-corrected reads of insert (ROIs) by using the Iso-Seq pipeline with minFullPass = 3 and minPredictedAccuracy = 0.9. Subsequently, full-length non-chimeric (FLNC) transcripts were identified by searching for the polyA tail signal and the 5’ and 3’ cDNA primers in ROIs. Iterative Clustering for Error Correction (ICE) was used to obtain consensus isoforms, and FL consensus sequences from ICE were polished using Quiver. High-quality FL transcripts were classified on the basis of post-correction accuracy of above 99%. Iso-Seq high-quality FL transcripts were used for removing redundancy by using cd-hit (identity > 0.99).

TransDecoder 5.5.0 (https://github.com/TransDecoder/TransDecoder/releases) was used to identify the candidate coding regions in transcript sequences in accordance with the following criteria: (1) the identification of a minimum length open reading frame (ORF) in the transcript sequence, (2) a log-likelihood score similar to that computed using the GeneID software of > 0, (3) the greatest score is obtained when the ORF is scored in the 1st reading frame compared with scores in the other five reading frames, (4) if a candidate ORF is fully encapsulated by the coordinates of another candidate ORF, the longer one is reported; however, a single transcript can report multiple ORFs (allowing for operons, chimeras, etc.), and (5) the putative peptide matches to a Pfam domain above the noise cutoff score (optional).

Four computational approaches, CPC, CNCI, CPAT, and Pfam, were combined to sort non-protein coding RNA candidates from putative protein-coding RNAs in the transcripts. Putative protein-coding RNAs were filtered out by using a minimum length and exon number threshold. Transcripts with lengths of more than 200 nt and more than two exons were selected as lncRNA candidates. These candidates were further screened using CPC/CNCI/CPAT/Pfam to distinguish protein-coding genes from non-coding ones.

Iso-Seq data were directly used to run all-vs-all BLAST with high-identity settings. BLAST alignments that met all criteria were considered products of candidate alternative splicing (AS) events. The alignment should contain two high-scoring segment pairs (HSPs) with the same forward/reverse direction. Within the same alignment, one sequence should be continuous or with a small overlap size (smaller than 5 bp), and the other one should be distinct to exhibit an AS Gap. The continuous sequence should completely align with the distinct sequence. The AS Gap should be larger than 100 bp and located at least 100 bp away from the 3'/5' end.

iTAK software [85] was used to predict plant transcription factors (TFs). TF identification and classification were based on the consensus rules (required and forbidden protein domains of each gene family) mainly summarized from PlantTFDB v5.0 [86] and by using families from AtTFDB [87] and PlantTFcat [88] as supporting evidence.

### Differentially expressed genes analysis

Gene expression levels were estimated in terms of fragments per kilobase of transcript per million fragments mapped (FPKM) by using RSEM v1.3.3 software [89]. Prior to differential gene expression analysis, the read counts were adjusted for each sequenced library by using the edgeR program package (3.16) [90] through one scaling normalized factor. Differential expression analysis of two samples was performed using the EBSeq R package [91]. The resulting false discovery rate (FDR) was adjusted using the posterior probability of being DE (PPDE). The FDR < 0.05 and |log2 (FC) |≥ 1 were set as the threshold for the identification of significant differential expression. The differentially expressed genes (DEGs) were subjected to enrichment analysis of GO and KEGG pathways.

### RT-qPCR validation

RT-qPCR was performed to validate the transcriptome data. Gene-specific oligonucleotide primers were used to select 18 candidate genes with FPKM ≥ 10 and read counts ≥ 20 in the RNA samples (Table S11). For reverse transcription, 2 µg mRNA and TUREscript 1^st^ stand cDNA Synthesis Kit (Aidlab Biotechnologies Co., Ltd, Beijing, China) were used. qRT-PCR was performed using 2XSYBR Green Master Mix (Bimake, Munich, Germany) on qTOWER 2.2 Quantitative Real-Time PCR Thermal Cyclers (Analytikjena, Jena, Germany). All samples were normalized to *Actin7* (F01_transcript_63797) to analyze the candidate gene expression level. The final relative expression levels of these candidate genes were calculated using the 2^−ΔΔCt^ method [92]. Three biological and technical replicates were considered for each treatment in all experiments. Error bars were used to represent the standard error of the mean. The Student’s *t*-test was used to compare the means of groups, with *p* < 0.05 considered statistically significant.

### Supplementary Information

**Additional file 1:** **Figure S1.** The predicted length distribution of the complete-CDS-encoded protein; **Figure S2.** The Nr annotated species taxonomic statistical map of all transcripts; **Figure S3.** GO categories for all transcripts in the transcriptome; **Figure S4.** KOG functional classification for the transcriptome sequences; **Figure S5. **(A-R) The expression profles (FPKM) in the transcriptome and qRT-PCR results of 18 candidate genes. (S) The correlation between qRT-PCR and the transcriptome data, as shown by values of log_2_(RPKM ratios) obtained by RNA-seq (x-axis), plotted against the values of log_2_(relative expression ratios) obtained by RT- qPCR (y-axis) for the 18 candidate genes. Each sample was analyzed in three biological replicates (significant differences at* p* < 0.05).

**Additional file 2:** **Table S1.** Sequencing data evaluation statistical table; **Table S2.** Candidate alternative splicing (AS) events prediction; **Table S3.** The annotation results of lncRNAs and lncRNA-targeted transcripts prediction; **Table S4.** lncRNA and the target transcripts associated with lignin, hemicellulose and cellulose biosynthesis; **Table S5.** The prediction of transcription factors; **Table S6.** KEGG pathway enrichment analysis of all transcripts; **Table S7.** The annotation information of the transcripts involved in the pathway of ko00940; **Table S8.** GO enrichment analysis of all DEGs; **Table S9.** KEGG pathway enrichment analysis of all DEGs; **Table S10.** Details information of the tree samples; **Table S11. **Details of the primers for the 18 candidate genes.

## Data Availability

All raw transcriptome sequences were deposited to the Sequence Read Archive at the National Center for Biotechnology Information (NCBI) with the accession number of SRP425632.

## Abbreviations

ABA: Abscisic acid
AIP: Auxin induced protein
ARF: Auxin response factor
AS: Alternative splicing
AUX: Auxin transporter protein
BP: Biological process
BRs: Brassinosteroids
CC: Cellular component
CCS: Circular consensus
CDS: Coding region sequence
CesA: Cellulose synthase
CKX: Cytokinin dehydrogenase
CLE: CLAVATA3/ESR
CLV: Leucine-rich repeat receptor-like protein
COG: Cluster of Orthologous Groups
CSL: Cellulose synthase-like
CTK: Cytokinin; DBH: diameter at breast height
DEGs: Differentially Expressed Genes
*E. urograndis*: *Eucalyptus urophylla* × *E. grandis*
E2: Ubiquitin-conjugating enzyme
ESS: Endogenous stress-related signal
ET: Ethylene
FC: Fold change
FDR: False discovery rate
FLNC: Full-length non-chimeric
FPKM: Fragments per kilobase of exon model per million mapped fragments
GA: Gibberellin
GID1B: Gibberellin receptor
GO: Gene ontology
GRP: Gibberellin-regulated protein
HPt: Histidine-containing phosphotransfer protein
HSPs: High-scoring segment pairs
ICE: Iterative clustering for error correction
INV: Acid beta-fructofuranosidase
KEGG: Kyoto encyclopedia of genes and genomes
KNOX: KNOTTED HOMEOBOX
KOG: EuKaryotic ortholog group
MF: Molecular function
NCBI: National center for biotechnology information
NR: Non-redundant
ORF: Open reading frame
PAL: Phenylalanine ammonia-lyase
PIN: Pin-formed proteins
PPDE: Posterior probability of being DE
PRR: Two-component response regulator-like
PYR: Abscisic acid receptor
REV: REVOLUTA
ROIs: Reads of insert
RPK: LRR receptor-like serine/threonine-protein kinase
RT-qPCR: Real time quantitative reverse transcription polymerase chain reaction
SERK: Somatic embryogenesis receptor kinase
STP: Sucrose transport protein
Susy: Sucrose synthase
TFs: Transcription factors
VND6 and VND7: Vascular-related NAC domain 6/7
WOX: WUSCHEL-related homeobox
