# Supplementary material for: Genes expression profiles in vascular cambium of Eucalyptus urophylla × Eucalyptus grandis at different ages
Source: BMC Plant Biol. 2023 Oct 18;23:500. doi: 10.1186/s12870-023-04500-8 (PMC10583469; doi:10.1186/s12870-023-04500-8)
Supplement: Supplementary file 1 — Additional file 1: Figure S1. The predicted length distribution of the complete-CDS-encoded protein; Figure S2. The Nr annotated species taxonomic statistical map of all transcripts; Figure S3. GO categories for all transcripts in the transcriptome; Figure S4. KOG functional classification for the transcriptome sequences; Figure S5. (A-R) The expression profles (FPKM) in the transcriptome and qRT-PCR results of 18 candidate genes. (S) The correlation between qRT-PCR and the transcriptome data, as shown by values of log2(RPKM ratios) obtained by RNA-seq (x-axis), plotted against the values of log2(relative expression ratios) obtained by RT- qPCR (y-axis) for the 18 candidate genes. Each sample was analyzed in three biological replicates (significant differences at p < 0.05). [file 12870_2023_4500_MOESM1_ESM.docx]

Additional file 1

Genes expression profiles in vascular cambium of *Eucalyptus urophylla* × *Eucalyptus grandis* at different ages

**Guo Liu^1,2^, Zhihua Wu^2^, Jianzhong Luo^1,2^, Chubiao Wang^2^, Xiuhua Shang^2^, Guowu Zhang^2*^**

1 State Key Laboratory of Tree Genetics and Breeding aboratory, Chinese Academy of Forestry, Beijing, China;

2 Research Institute of Fast-growing Trees, Chinese Academy of Forestry, Zhanjiang, China;

* corresponding author: fyzgwu@163.com


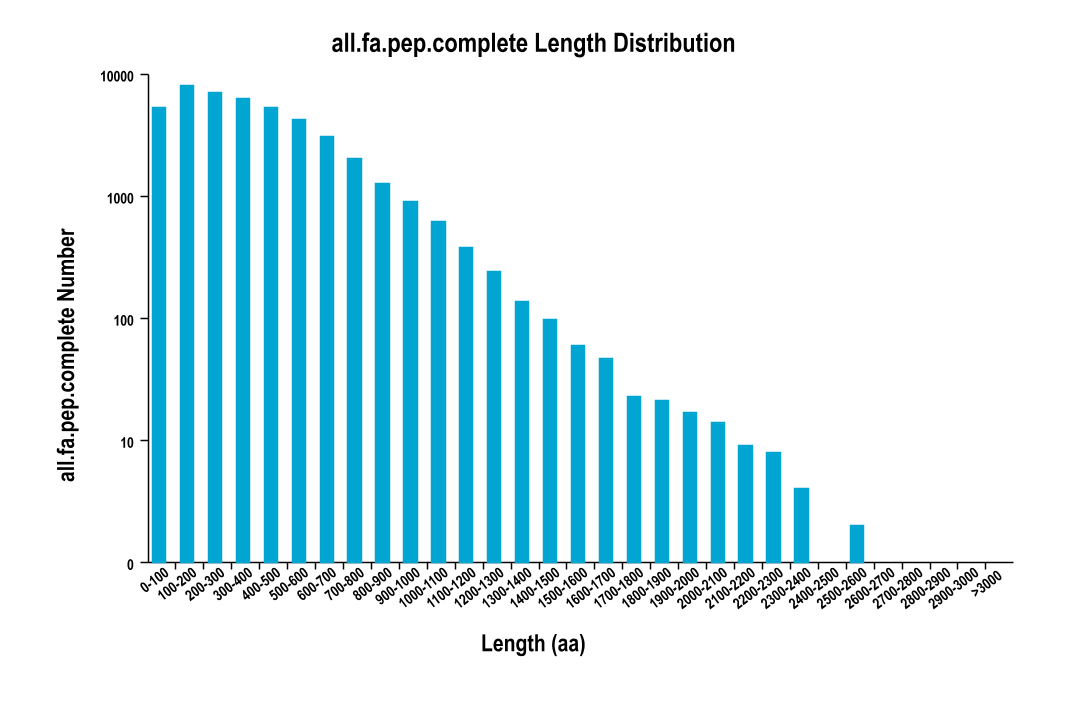


**Figure S1.** The predicted length distribution of the complete-CDS-encoded protein.


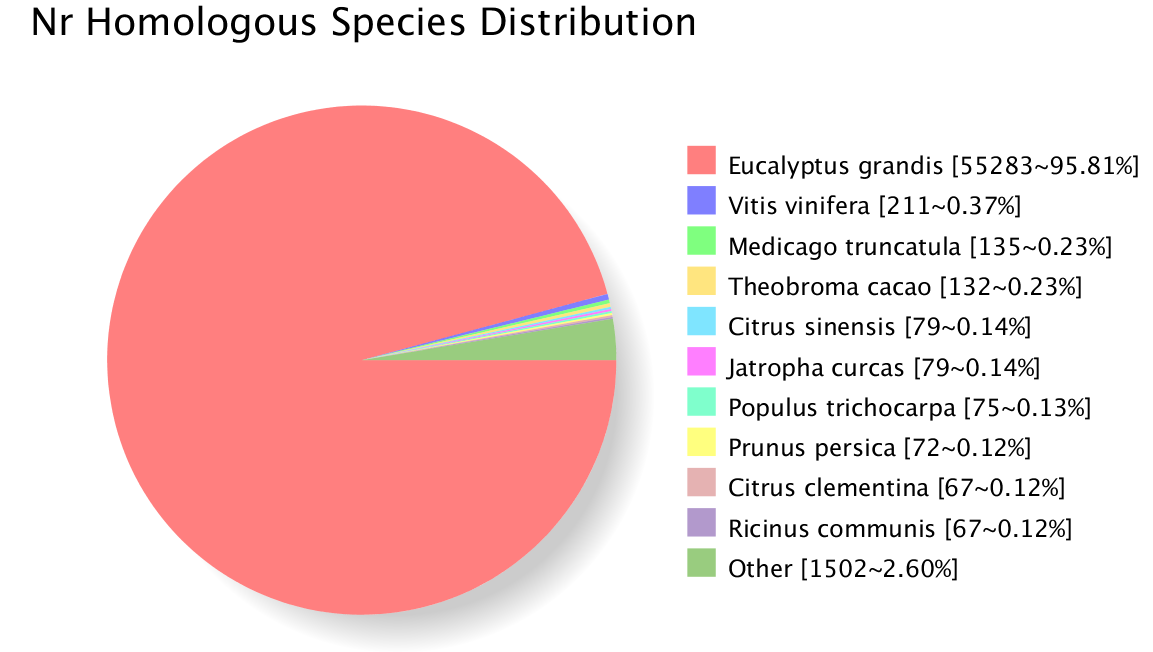


**Figure S2.** The Nr annotated species taxonomic statistical map of all transcripts.

Note: Different colors represent different species.


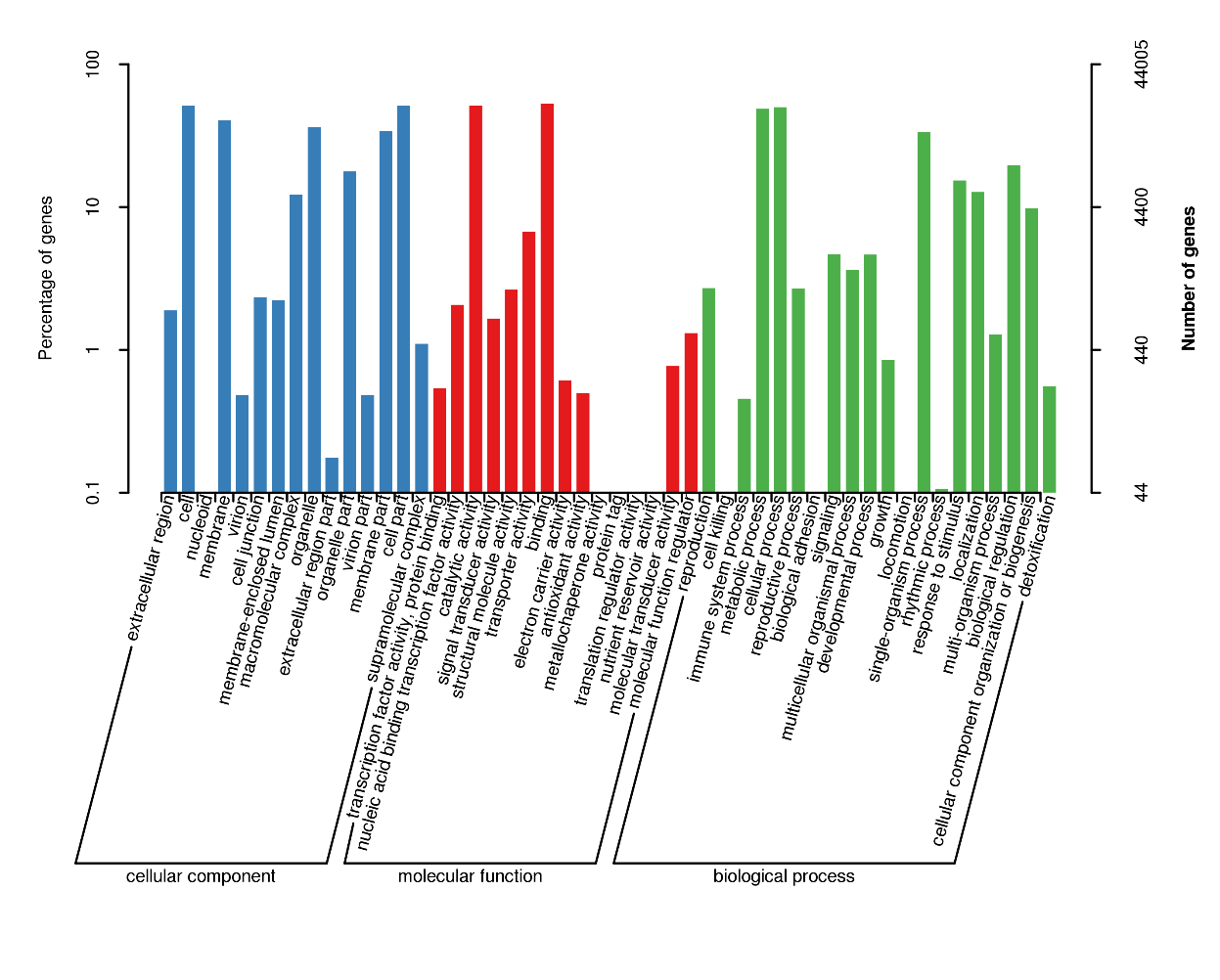


**Figure S3.** GO categories for all transcripts in the transcriptome.

The right y-axis represents the number of transcripts in a category and the left y-aixs represents the percentage of transcripts in a category.


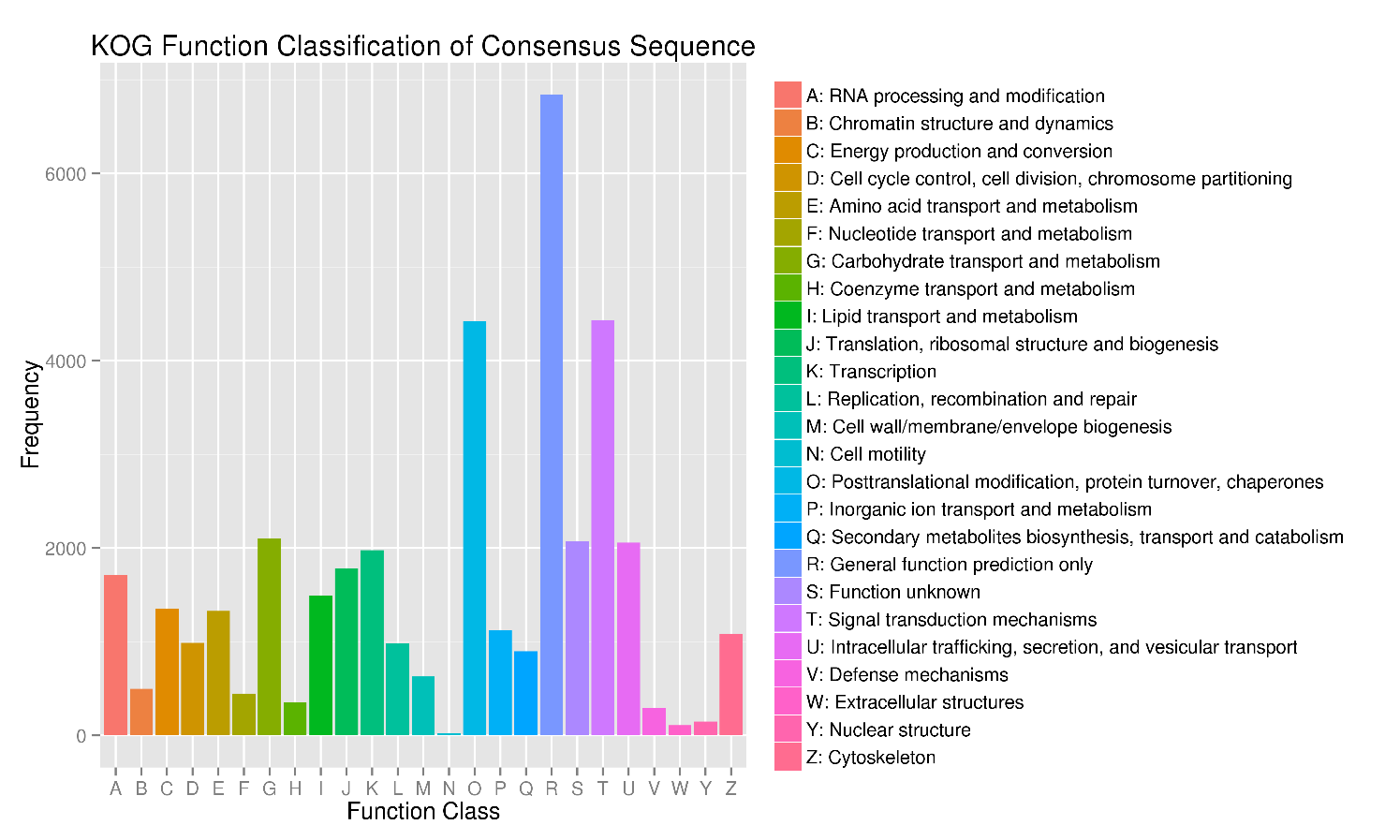


**Figure S4.** KOG functional classification for the transcriptome sequences.


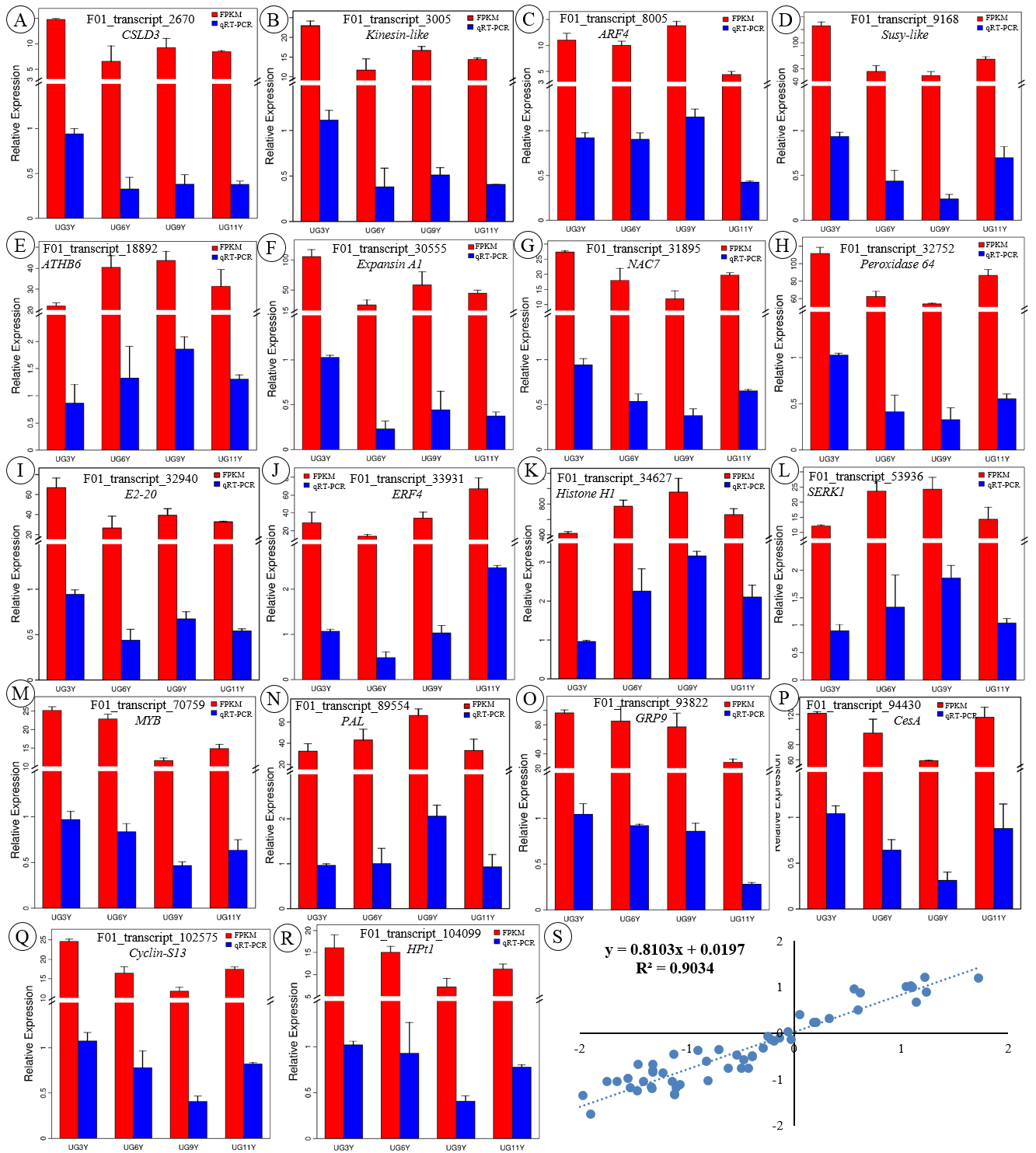


**Figure S5.** (A-R) The expression profles (FPKM) in the transcriptome and RT-qPCR results of 18 candidate genes. (S) The correlation between qRT-PCR and the transcriptome data, as shown by values of log_2_(RPKM ratios) obtained by RNA-seq (x-axis), plotted against the values of log_2_(relative expression ratios) obtained by RT-qPCR (y-axis) for the 18 candidate genes. Each sample was analyzed in three biological replicates (significant differences at *p* <0.05).
